# Extensive injuries following a ‘trip at home’: a case report

**DOI:** 10.4076/1757-1626-2-8303

**Published:** 2009-08-26

**Authors:** Maruthesh Gowda Chikkappa, Charles Morrison, Andrew Lowe, Jay Gokhale, Ralph Antrum

**Affiliations:** 1 Department of General Surgery, Bradford Royal Infirmary, BradfordBD9 6RJUK; 2 Department of Radiology, Bradford Royal Infirmary, BradfordBD9 6RJUK

## Abstract

A 52-year-old, Caucasian, British man suffered significant injury following simple fall. A man with no significant past medical history, presented to the accident and emergency with right side chest pain and shortness of breath. He reported a simple fall, two days before admission. Chest radiograph showed simple bilateral pneumothorax and pneumomediastinum. Subsequent computerised tomography confirmed the thoracic injury and identified complex pathophysiology as described. This case shows the extent of injury a person can sustain from a simple fall and the high index of suspicion required to discover the full extent of a patient's injuries. We review the literature to find other forms of presentation.

## Case presentation

A previously fit and well 52-year-old, Caucasian, British man presented to accident and emergency two days after tripping and falling on to his right side at home. On arrival, patient complained of shortness of breath and right side chest pain. He was found to be in obvious distress, dyspnoeic, restless and haemodynamically unstable with blood pressure of 86/64, respiratory rate 32, saturation 85%, pulse 120 bpm and temperature was 36. He was in pain but airway was maintained. Extensive emphysema from neck to scrotum was noted.

**Figure 1.**
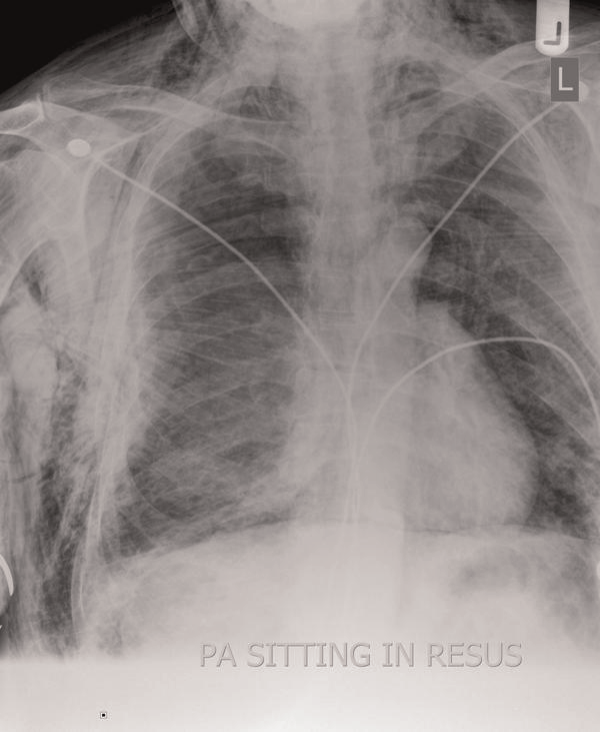
CXR showing bilateral pneumothoraces.

The patient was resuscitated in accordance with Advanced Trauma and Life Support (ATLS) guidelines and was given high flow oxygen, intravenous fluids and was attached to a cardiac monitor. Chest examination revealed bilateral reduced air entry and tenderness over the right 5^th^, 6^th^ and 7^th^ ribs. Trachea was central and normal cardiac sounds were heard. Immediate chest radiograph (Figure 1) showed fracture of right 5^th^, 6^th^ and 7^th^ ribs but no flail segment and bilateral pneumothoraces which were treated with bilateral chest drains with under water seals. ECG done at the time was normal. Abdomen was soft and non tender with normal bowel sounds.

Blood tests revealed C reactive protein of 57, and leukocyte count of 21.16 with neutrophilia of 18.95.

**Figures 2.**
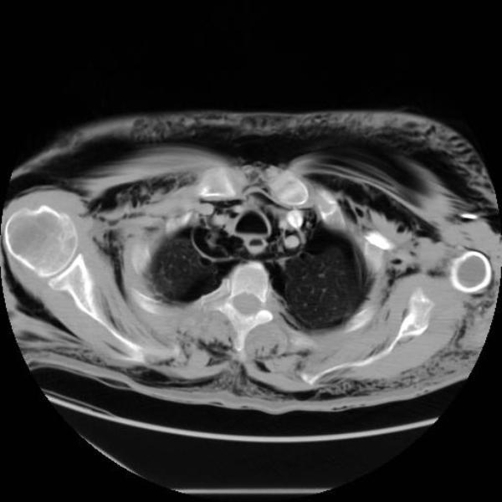
CT showing rib fractures.

**Figures 3.**
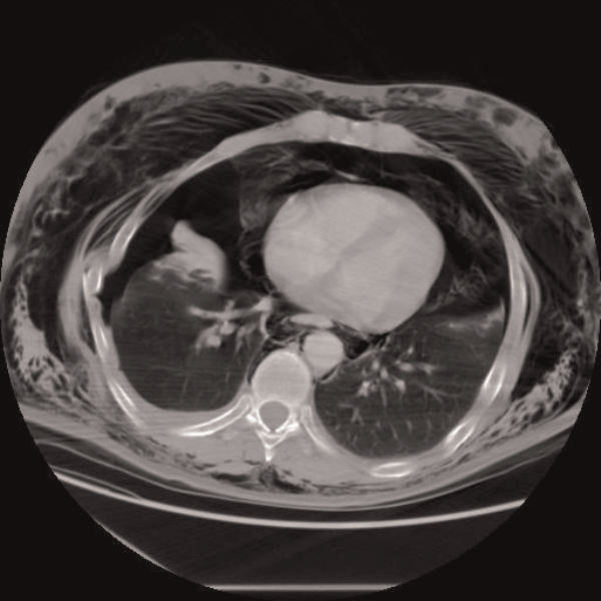
CT showing bilateral pneumothoraces.

**Figures 4.**
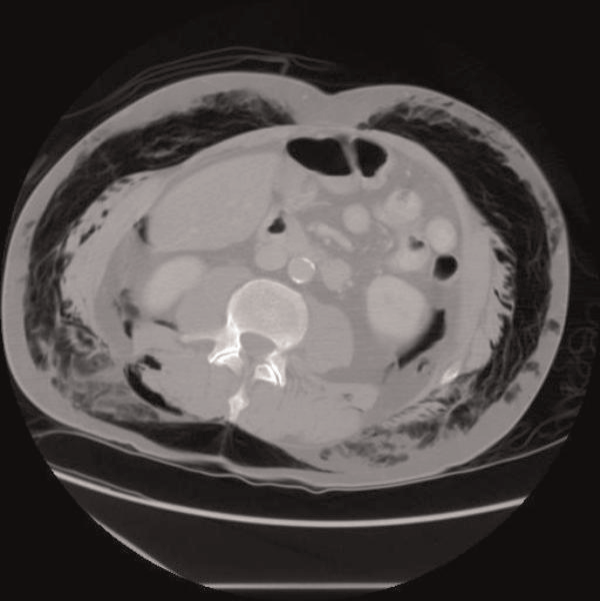
CT showing retropneumoperitoneum.

To assess the full extent of injury, the patient underwent computerised tomography (CT) of chest, abdomen and pelvis which confirmed right sided rib fractures (5^th^, 6^th^ and 7^th^) (Figure 2) and right pneumothorax, leading to bronchopleural subcutaneous fistula leading to left pneumothorax (Figure 3). Emphysema from neck to lower part of scrotum, pneumomediastinum and retropneumoperitoneum (Figure 4) (right retroperitoneal space around ascending colon and right kidney) were also reported.

The patient was admitted for supportive care, analgesia and physiotherapy. Pneumothoraces resolved after 5 days and chest drains were removed. By 7^th^ day subcutaneous emphysema had reduced significantly and the patient was discharged home. On subsequent review in clinic all the subcutaneous emphysema had resolved and patient was asymptomatic.

## Discussion

The combination of injuries following simple fall reported in this case has not been described in the literature previously. This degree of widespread gas tracking has been reported after iatrogenic perforation following endoscopic examination of colon [1-3], and in all these cases there was insufflation of gas through perforated colon and gas has travelled from abdomen to thorax along a pressure gradient.

Intra-abdominal pressure exceeds intrathoracic pressure by 20-30 cms of H_2_0 during both inspiration and expiration [2]. For this reason, simple pneumothorax normally does not lead to retropneumoperitoneum. Even patients with tension pneumothorax do not develop retropneumoperitoneum because they are either treated rapidly or intrathoracic pressure never rises above intra-abdominal pressure [3].

Grosfled [4], reported in cats, when intra-tracheal pressure exceeds intra-abdominal pressure by 40 cms H_2_0 it leads to interstitial emphysema. When pressure exceeded 50 cms of H_2_0, pneumoperitoneum occurred. Only at pressures above 60 cm of H_2_0 resulted in both subcutaneous emphysema and pneumoperitoneum. This experimental data suggests that a significant pressure gradient is required to force air to track from the thoracic cavity to abdominal cavity. It is thought that air which dissects from ruptured alveoli can travel along vessels to mediastinum and with further increase in pressure dissect along pleural space, along great vessels and the oesophagus. This air then dissects into retropneumoperitoneum.

The published cases which describe a similar combination of injuries have all been associated with a far greater trauma than that is described in this report [5].

This case demonstrates the necessity of high index of suspicion to ensure that the full extents of patient's injuries are ascertained, even where mechanism of injury appears to be trivial.
